# Why Metacognition Is Not Always Helpful

**DOI:** 10.3389/fpsyg.2020.01537

**Published:** 2020-07-02

**Authors:** Elisabeth Norman

**Affiliations:** ^1^Department of Psychosocial Science, Faculty of Psychology, University of Bergen, Bergen, Norway; ^2^Faculty of Health Sciences, UiT The Arctic University of Norway, Tromsø, Norway

**Keywords:** metacognition, normative, metacognitive knowledge, metacognitive strategies, introspection, verbalization, metacognitive feelings, mindlessness–mindfulness

## Abstract

In many situations, actively engaging in metacognition may improve cognitive achievement and subjective well-being. However, the potential disadvantages of metacognitive engagement are only rarely communicated in metacognition research. In this paper, I outline three ways in which metacognition may reduce cognitive achievement and psychological well-being. First, metacognition may sometimes actively interfere with task performance. Second, the costs of engaging in metacognitive strategies may under certain circumstances outweigh its benefits. Third, metacognitive judgments or feelings involving a negative self-evaluation may detract from psychological well-being. The main contribution of this paper is to integrate findings from different research traditions in order to illustrate the three suggested ways in which metacognition may be unhelpful. An implication of this overview is that although metacognition is most often beneficial to cognitive achievement and subjective well-being, one should bear in mind that it may also have the opposite effect. It is important for researchers and practitioners to take this potential downside of metacognition into account. Practitioners might find it useful to consider the following three questions that relate to my aforementioned claims: Is the nature of the task such that metacognition could interfere with performance? Is the cognitive demand required by the metacognitive strategy disproportionally large compared to its potential usefulness to cognitive achievement? Does metacognition lead to an unhelpful comparison of oneself to others? The same considerations should be kept in mind when researchers and practitioners communicate the potential implications of research findings in metacognition research to audiences within and beyond the research community.

## Introduction

Metacognition is a hot research topic. A vast number of scientific papers and books have addressed metacognition since the concept was first introduced in the 1970s (Flavell and Wellman, 1977; Flavell, 1979), and a variety of methodological approaches have been developed and applied within different psychological subdisciplines (Norman et al., 2019). Metacognition has also become an increasingly popular term in everyday language, and is frequently used in, for instance, educational settings (Dimmitt and McCormick, 2012).

Researchers commonly refer to metacognition as consisting of three facets (Flavell, 1979). *Metacognitive knowledge* refers to people’s general knowledge and understanding of various cognitive processes, and of their own versus other people’s cognitive abilities and strategies (Efklides, 2008). *Metacognitive strategies* are deliberate strategies used to control cognition (Efklides, 2008). *Metacognitive experiences* are feelings, judgments, and task-specific knowledge that reflect what the person is aware of and feels during task performance (Efklides, 2008).

Metacognition research often seems concerned with what people *ought* or *ought not* to do. I first provide a tentative description of this *normative aspect* of metacognition research, suggesting that the majority of metacognition research gives more attention to the potential benefits of metacognition than to its potential disadvantages. Then I present examples of specific ways in which metacognition is sometimes *not* beneficial to cognitive achievement and psychological well-being. In my opinion, these have not yet been given adequate voice in the literature, and there have not been many attempts to integrate examples of negative influences of metacognition from different research traditions. This paper attempts to provide such an integration and to thereby increase awareness of the potential downside of metacognition. The main focus is on *metacognitive strategies*, which is the facet most often associated with a conscious choice. However, to the extent that the three facets are interrelated (e.g., applying a metacognitive strategy would most often require activating metacognitive knowledge and experiencing metacognitive feelings), all three will be addressed.

## Normative Aspects of Metacognition Research

Most would agree that metacognition serves to *monitor* and *control* ongoing cognitive activity (Nelson and Narens, 1990). Metacognition as a research topic has spread to multiple areas of psychology, including developmental, personality, social, clinical, and forensic psychology, to mention a few. Thus, it has become a cross-disciplinary subject (Koriat, 2002, 2007). Also across these subdisciplines, the functional role of metacognition is widely agreed upon (Norman et al., 2019).

Closely linked to this functional role is the idea of something that the individual *ought to* strive for, some goal that they should try to reach by applying metacognitive strategies or knowledge. Two candidate outcome variables can be outlined. One is *cognitive achievement.* This relates to how much the individual learns or remembers, how well the person solves problems, to what extent the person can make rational decisions and reason logically, etc. Metacognition could play a role in cognitive achievement by helping the person make use of the best cognitive and metacognitive strategies in the given situation.

The other category of outcome variable is *psychological well-being*. Some definitions of well-being focus on happiness, life satisfaction, and positive affect (Diener, 1984) some on the absence of distress and dysfunction (see Joseph and Wood, 2010 for a critical perspective), and yet others on the balance between the individual’s challenges and resources (Dodge et al., 2012). I suggest that metacognition could influence well-being in at least two ways. One is through its influence on cognitive achievement. This would happen in cases where metacognitive activity improves cognitive achievement and thus leads to positive experiences. For instance, applying metacognitive strategies may help a student achieve a higher grade, which makes the student happy. The other is through the metacognitive activity itself being subjectively experienced as pleasant or unpleasant, which could directly affect a person’s current mood and well-being. For example, a strong feeling of comprehending a text could be experienced as positive. A contrasting feeling of low comprehension could be experienced as negative.

## Metacognition as Helpful

In some parts of the literature, the normative ideal of increasing metacognitive awareness is explicitly stated. For example, educational programs inspired by metacognition literature often encourage teachers to increase students’ metacognitive awareness and abilities to improve learning (Siegesmund, 2016) or social skills and psychological well-being (Umino and Dammeyer, 2016). The importance of metacognitive awareness is also highlighted in the clinical literature on metacognitive therapy (Wells, 2011) which assumes a role of patients’ metacognitive awareness of dysfunctional cognitive patterns—together with acquisition of alternative metacognitive strategies—in recovery from mental illness. In medical decision making, Stark and Fins (2014) have argued for medical professionals’ “ethical imperative to thinking about thinking” in order to prevent diagnostic errors.

However, also when this is not explicitly stated, metacognition research often seems to imply that metacognitive activity is beneficial. Being metacognitively active could involve being aware of metacognitive beliefs and knowledge and actively applying metacognitive strategies. As metacognition has received increased popularity in psychological disciplines, the impact of this normative assumption has spread correspondingly. Even when researchers express no opinion on whether metacognition is useful, empirical findings are often used to argue for its beneficial effects. This also extends beyond the research community. For example, the term metacognition is often used by teachers and school leaders in Norway, where children as young as 6–7 are encouraged to apply it in their own learning (Fleming et al., 2010; Furnes and Norman, 2016). The widely held assumption that metacognition is beneficial, could at least in part be understood as a consequence of its close relationship to self-regulation (Zimmerman, 2008; Efklides, 2011). Thus, the benefits of metacognition could be inferred from literature that has argued for the benefits of willpower, also in contexts beyond education (Baumeister and Tierney, 2012).

## Metacognition as Unhelpful

There are also examples of research showing how metacognition can sometimes be unhelpful. Because such research findings may provide guidance as to when one should encourage or discourage metacognitive activity, it is important that they are communicated to both researchers and practitioners. Yet, such findings are rarely given much weight in the metacognition literature. One reason could be that some of this research has not been conducted under the heading of “metacognition,” even when clearly addressing metacognitive phenomena. Therefore, these findings are rarely discussed cojointly. Another reason could be that common opinion is that all in all, the potential disadvantages of metacognition are less important because they are normally outweighed by its advantages.

However, disadvantages of metacognition may still be important. In the following, I therefore summarize some relevant findings from different research areas, exemplifying ways in which metacognition can sometimes *not* be helpful—or even outright *unhelpful—*to cognitive achievement and psychological well-being. The discussion will center around three suggestions, namely, that (1) metacognition may actively interfere with task performance, (2) the costs of engaging in metacognitive strategies may outweigh the benefits, and that (3) metacognitive judgments or feelings involving a negative self-evaluation may detract from psychological well-being^1^. I address all three facets of metacognition.

### Metacognition May Actively Interfere With Task Performance

I some situations, certain forms of metacognition may actively interfere with task performance.

One example is concurrent verbalization of metacognitive experiences. There is empirical evidence to show that concurrent explanations of metacognitive experiences can impair performance, at least when it comes to experience-based, “intuitive” feelings. In a series of findings by Schooler et al. (1993, 1997), the negative effect of verbalization on cognitive task performance is commonly referred to as “verbal overshadowing” (see also Yamada, 2009). Verbal overshadowing has been explained in terms of a *discrepancy* between verbal labels and properties of the perceptual experience, a *processing shift* from global to local processing, and a *criterion shift* toward more conservative responding (Chin and Schooler, 2008). In contrast, others have demonstrated that verbalization in some situations can be helpful and improve performance (Leisti et al., 2014). Of course not all verbalizations involve metacognition. These are limited to those instances where the person attempts to verbalize some aspect of a cognitive process or its outcome. Examples include descriptions of sensory experiences, problem solving, and subjective preferences.

A different line of arguments suggesting that attention to metacognitive experiences is not always beneficial, comes from research on *mindlessness* (Neal et al., 2011). Simply defined, mindlessness is the opposite or absence of mindfulness. Langer (1992) defines mindlessness as lack of attention or presence, resulting in or caused by automatic application of existing knowledge. Thus, it is characterized by *cognitive inflexibility.* Di Nucci (2013) describes mindlessness as being characterized by automated and unconscious processing, or what we associate with “System 1” thinking, i.e., fast, automatic/uncontrollable, effortless, associative, implicit, and emotionally charged thinking (Kahneman, 2003). Whereas some regard mindlessness as a state that should be avoided (Langer, 1992) others have argued that mindlessness may at times be beneficial (Di Nucci, 2013; Kashdan and Biswas-Diener, 2014). For instance, complex decisions that largely involve implicit/unconscious knowledge may best be made mindlessly. Mindfulness can be seen as a form of metacognition (Shapiro et al., 2006; Jankowski and Holas, 2014). Thus, in the same way that mindlessness can sometimes be beneficial, the choice to not execute a metacognitive strategy or to ignore a metacognitive experience could sometimes benefit cognitive performance.

A third example is overconfidence. Studies of the Dunnig–Kruger effect (Kruger and Dunning, 1999) have shown that people whose performance falls within the lower quartile on various laboratory and real-world tasks, tend to overestimate their performance relative to people who perform better. This has been referred to as a “double curse” (Dunning, 2011) because it appears that the same shortcomings responsible for low performance also prevents low-performing individuals from recognizing that they are making errors. It could be argued that in cases where learning and improvement are unlikely to occur, overconfidence may be beneficial to the person’s self-image and mood. However, this may not represent the whole truth. Even when learning is unlikely, there may be potential downsides to overconfidence. For example, someone who overestimates their abilities may invest their cognitive resources in a non-optimal fashion. Moreover, unrealistically high expectations of oneself that are not fulfilled may cause distress^2^. Thus, it is not difficult to imagine potential negative long-term effects of overconfidence.

### The Costs of Engaging in Metacognitive Strategies May Outweigh the Benefits

Metacognitive strategies are people’s deliberate attempts to control cognition by applying various learned skills. For instance, adequate reading comprehension may require being able to adapt one’s reading speed to the complexity of the text, and to go back and repeat difficult words or sentences if comprehension is low. Metacognitive strategies are generally seen as important both in student learning and in other cognitively demanding situations. In clinical psychology, metacognitive strategies refer to the monitoring and control of thoughts related to a mental disorder. This includes both learned, unhealthy thought patterns that contribute to the problem, and learned behaviors used to break those patterns. Imagine a patient with generalized anxiety. Certain metacognitive strategies could contribute to this condition. These include the constant monitoring of thoughts and threats, thought suppression, and checking behavior. Therapeutic metacognitive strategies might include keeping track of the time is spent on compulsive checking, and prioritizing and planning ahead without rigidity (Sudhir et al., 2017).

The aforementioned cases of verbalization and mindlessness exemplify how the specific costs of engaging in particular forms of metacognitive strategies in specific cases may outweigh its benefits. Additionally, engaging in metacognitive strategies may come at a more general cost: The intentional application of a metacognitive strategy could be demanding in terms of time and cognitive resources. Metacognitive strategies must be learned, either through explicit instruction or implicitly through everyday experiences. This is the case both for those strategies that address purely cognitive tasks like reading or problem solving, and for strategies aimed at improving mental health. Although a strategy can become largely automatized with extended practice, the implementation of most strategies is likely to require some degree of initiative or effort. In many cases, applying metacognitive strategies may obviously be helpful and improve cognitive performance and/or well-being. However, in cases where it is not so, going ahead with a cognitive task *without* employing an effortful metacognitive strategy could lead to higher subjective well-being simply because it would be less straining/demanding. For example, if reading a novel was part of a student’s course requirement in English, a conscious strategy to monitor one’s comprehension during reading is unlikely to increase comprehension, but could very well reduce well-being. In such cases, it could be argued that encouraging people to acquire and use metacognitive strategies would not always be helpful.

The idea that the cost of engaging in metacognitive strategies may outweigh its benefits has some parallels to the concept of bounded rationality in decision making (Simon, 1957).

### Metacognitive Judgments or Feelings Involving a Negative Self-Evaluation May Detract From Psychological Well-Being

Metacognitive beliefs (i.e., a form of metacognitive knowledge) may address a person’s evaluation of their own abilities and self-worth (Tarricone, 2011). For instance, a person may—correctly or incorrectly—assume that they are less able/talented than other people when it comes to some cognitive ability. This could in turn lower the person’s self-esteem and self-efficacy and thereby reduce their efforts in and motivation for trying to do their best on a certain cognitive task. Believing that others are more gifted or able than oneself in a certain area could have a similar effect. Metacognitive beliefs could also take the form of ideas about how one should *ideally* be or behave. For example, a high school student could mistakenly believe that one should ideally learn all central definitions of a certain subject by heart. To the extent that such beliefs represent distorted or exaggerated views of reality, they may hinder successful adaptation. This might in principle impair both cognitive achievement and psychological well-being. For example, the mistaken belief of the student in our previous example may increase stress and reduce motivation, which could impair the student’s achievement on the exam, and reduce well-being.

## Metacognition as Unhelpful: Some Clarifications

I started off by suggesting that metacognition research has a normative side. Empirical and theoretical research on metacognition often seems to imply that metacognitive sensitivity, metacognitive awareness, and the active use of metacognitive knowledge and strategies are helpful and something we should strive for. In the paper I have attempted to show that this may not always be the case. I have given some brief examples of times when metacognition may hinder rather than facilitate performance, and reduce rather than increase psychological well-being. I have outlined some preliminary hypotheses. The analysis is tentative and needs to be followed up by empirical studies, although some already have empirical support. To date, claims (1) and (2) have more empirical support than claim (3).

Importantly, I am not trying to argue that people can always choose whether or not to “be metacognitive” in a given situation. Each facet of metacognition could in principle be activated automatically or voluntarily. For instance, metacognitive knowledge of ourselves (e.g., “I’m useless at quizzes”) can be activated involuntarily. At the same time, we can intentionally choose to retrieve and reflect upon the same knowledge. Similarly, even though a metacognitive experience (of, e.g., confidence) can sometimes occur regardless of one’s conscious intent, we can to a certain extent choose to attend to or ignore such experiences. The application of metacognitive strategies (e.g., to pay attention to how well you understand a text), could be seen as resulting from a conscious intention. However, the application of a metacognitive strategy (e.g., to read slower if comprehension is low) could also be largely automatic. Thus, the voluntary nature of metacognition primarily relates to the *activation* of metacognitive knowledge, the decision to *attend to* one’s metacognitive feelings, and the *intentional use* of metacognitive strategies. It should also be noted that some of my examples concern what may be labeled “good” metacognition whereas others concern “bad” metacognition (i.e., false metacognitive conclusions).

My examples are taken from research on normally functioning individuals. However, there are also some obvious clinical implications. Well-being can, for example, be affected in cases where a person is too sensitive to metacognitive feelings. One such example is obsessive-compulsive disorder (OCD). According to Coles et al. (2003), OCD is characterized by an increased tendency to experience and attend to “not just right” experiences. Thus, being metacognitively sensitive might be beneficial, but in some contexts only up to a certain point. As pointed out by Janeck et al. (2003), the tendency to engage in excessive amounts of metacognitive self-reflection “may increase opportunities for negative appraisals of intrusive thoughts, foster over-importance of thought beliefs, and increase the likelihood of developing OCD” (ibid., p. 181).

## Concluding Remarks

Metacognition is a normal part of cognitive functioning. We cannot choose to “be metacognitive” or not. However, we can choose whether to apply certain metacognitive strategies, attend to metacognitive feelings, or reflect upon metacognitive knowledge. In various clinical and educational settings, this is often encouraged (Wells, 2011; Siegesmund, 2016; Umino and Dammeyer, 2016). Here, metacognition implies something more than experiencing naturally occurring metacognitive activity. An increased focus on metacognition could perhaps be seen as related to the more general therapeutic self-help culture (Madsen, 2015) and my claims consistent with critical perspectives to this trend.

I have shown that metacognition can be unhelpful in at least three ways. Correspondingly, before encouraging someone to engage in metacognition, it is relevant to consider the following three questions: Is the nature of the task such that metacognition could interfere with performance? Is the cognitive demand required by the metacognitive strategy disproportionally large compared to its potential usefulness to cognitive achievement? Does metacognition lead to an unhelpful comparison of oneself to others? If the answer to any of these is yes, metacognition might be more unhelpful than helpful.

## Author Contributions

The author confirms being the sole contributor of this work and has approved it for publication.

## Conflict of Interest

The author declares that the research was conducted in the absence of any commercial or financial relationships that could be construed as a potential conflict of interest.

## Footnotes

1 I would like to thank a reviewer for suggesting this way of structuring my arguments.

2 I would like to thank a reviewer for raising this point.
